# Pharmacological Effects of *Hydrocotyle bonariensis* Comm. ex Lam (Araliaceae) Extract on Cardiac Activity

**DOI:** 10.1155/2022/1961352

**Published:** 2022-09-29

**Authors:** Kaboua Komla, Pakoussi Tcha, Mouzou Aklesso, Kadissoli Balakiyém, Assih Mindede, Bois Patrick, Chatelier Aurelien

**Affiliations:** ^1^Laboratory of Physiology-Pharmacology, FDS, University of Lomé, BP 1515, Lomé, Togo; ^2^Laboratory of Signaling and Membrane Ion Transport, Pôle Biosanté, University of Poitiers, BP 86000, Poitiers, France

## Abstract

*Hydrocotyle bonariensis* is one of the medicinal plants used in traditional medicine for the management of hypertension in Africa, Asia, and Latin America. However, the real impact of the traditional use of this plant on arterial hypertension has not yet been the subject of conclusive scientific information in the literature. This study aimed essentially to evaluate the potential cardiomodulatory effect of the hydroethanolic extract of *Hydrocotyle bonariensis*. In other to do so, the hydroethanolic extract of *H. bonariensis* was studied *in vivo* on the Wistar rat ECG and then *in vitro* on the isolated perfused Wistar rat heart using the Langendorff system. The extract was also tested on isolated guinea pig atria kept alive in the organ-specific vessel under physiological conditions similar to those of a living organism. At the cellular level, the effects of the extract were evaluated on the human cardiac sodium current INav1.5 and on the human cardiac pacemaker current If. We noted that the extract caused a decrease in P wave and T wave amplitudes and heart rate and an increase in the duration of the RR interval on the *in vivo* rat ECG. On the isolated perfused Langendorff heart as well as on the isolated atria, a decrease in the RR interval and in the heart rate was noted. The extract had no effect on human cardiac sodium current, but it did reduce human cardiac pacemaker current. In conclusion, the present study demonstrated that *Hydrocotyle bonariensis*, a medicinal plant traditionally used to prevent and treat hypertension, has an overall cardiomoderating effect. This effect would contribute to the reduction of blood pressure.

## 1. Introduction

Hypertension, a major cardiovascular risk factor, is known to shorten the lifespan of a significant proportion of the world's population and considerably increase the number of annual deaths. It currently affects 1.28 billion people worldwide and contributes to the development of other cardiovascular diseases (WHO, 2021). This pathology sets in when a failure occurs in the normal regulation of blood pressure [1, 2]. In addition to peripheral arterial resistance, cardiac output is one of the two critical factors targeted by the development of hypertension. Cardiac function, therefore, remains a justified fundamental target of certain drugs and cardiovascular pharmacological research related to hypertension. Despite the efforts against cardiovascular diseases, hypertension spread remains persistent and increasing, with a heavy prevalence rate in the future [3]. This situation obliges scientists to deepen cardiovascular research while diversifying the sources of candidate molecules such as phytotherapy. Indeed, more than 80% of the African or even global population have always resorted to plants to relieve themselves or to overcome their various pathologies, including hypertension [4]. *Hydrocotyle bonariensis*, a semiaquatic herbaceous plant of the Araliaceae family, has interesting ethnobotanical data regarding its antihypertensive virtues. According to Burkill [5] and Monyn et al. [6], *Hydrocotyle bonariensis* is used by traditional medicine practitioners to treat high blood pressure. Our previous work has demonstrated its antiarrhythmic cardiac effects [7] as well as its safety by oral administration [8]. In order to further evaluate the antihypertensive potential of this plant, we set the objective in this study to further investigate experimentally its effects on cardiac function. Specifically, we test the hydroethanolic extract of the leaves of this plant on the electrocardiogram, isolated perfused heart, isolated atria, cardiac sodium current, and mammalian cardiac pacemaker current.

## 2. Material and Methods

### 2.1. Preparation of *Hydrocotyle bonariensis* Leave Hydroethanolic Extract

The leaves of *Hydrocotyle bonariensis* were collected from the botanical garden of the Faculty of Sciences of the University of Lomé. These leaves were identified at the Laboratory of Botany and Plant Ecology of the Faculty of Sciences of the University of Lomé and deposited in the herbarium under the identification number TG 15183. After harvesting, they were washed abundantly with tap water and then dried under air-conditioning at 20°C in the dark.

After 3 weeks of drying, the leaves of *Hydrocotyle bonariensis* (Araliaceae) were pulverised, and then 320 grams was macerated for 72 hours with intermittent stirring in a hydroethanolic solvent in an equal volume ratio (1 : 1). After maceration, the solution was filtered through filter paper and cotton. The filtrate obtained was evaporated under vacuum using a Rotavapor at 45°C. 68.28 grams of the extract was obtained, giving a yield of 21.34%. The extract obtained was kept cool for the preparation of the different concentrations to be used for the tests.

### 2.2. Testing of *H. bonariensis* Extract on the Electrocardiogram (ECG) of Wistar Rats *In Vivo*

*Wistar* Albino rats weighing between 180 and 250 g and guinea pigs between 220 and 300 g were used. The animals were reared in the animal house of the Physiology-Pharmacology Laboratory of the Faculty of Sciences (University of Lomé) under standard light (natural day/night cycle of 12 h) and temperature (25°C). The rats were fed a standard diet. Principles of laboratory animal care as described in institutional guidelines and ethics of Laboratory of Physiology/Pharmacology of the University of Lomé, Togo (ref: 001/2012/CB-FDS-UL), were followed.

For electrocardiogram (ECG) recording, 16 male rats were anesthetized by intraperitoneal injection of sodium pentobarbital (Dolethal) 182 mg/kg and then fixed on the restraint table. The jugular vein was exposed and intubated with a thin polyethylene catheter attached to a syringe. The 0.9% heparinized 10% NaCl solution was injected at a rate of 0.1 ml/100 g body weight to prevent blood clotting. The axilla of both forelimbs and the groin of both hindlimbs were shaved and cleaned with alcohol. Four electrodes were attached at these sites according to the color/position relationship indicated in the recording system instructions [9]. ECG measurements were performed using an electrocardiogram transducer coupled to an acquisition system (PowerLab; ADInstruments, Castle Hill, NSW, Australia) connected to a computer driven by LabChart6.0 software (ADInstruments).

Rats were each administered with 0.1 ml/100 g body weight, 0.9% NaCl solution i.v. for control and hydroethanolic extract of *H. bonariensis* leaves diluted in 0.9% NaCl solution at 10, 20, 40, and 80 mg/kg i.v., respectively. The extract was administered to rats via the jugular vein at a dose of 0.1 ml/100 g after a blood pressure stabilization time of approximately 30 minutes. The effects of this extract on ECG were recorded continuously throughout the experiment.

### 2.3. Testing of *H. bonariensis* Extract on Isolated Perfused Wistar Rat Heart (*In Vitro*)

The study of the effects of the extract on isolated heart was performed with the inspiration of the method of Carré et al. [10]. Four male rats were anesthetized as before. The rat heart was harvested after checking for the absence of vegetative reflex (paw pinch) under deep anesthesia. The animal was placed on its back, its 4 limbs were attached to the dissection stand, and the ventral part of the rat was moistened with alcohol to prevent hair dispersion. The excised heart was rapidly immersed in a bath of Krebs–Henseleit solution cooled to 4°C in a Petri dish with a Sylgard bottom (Krebs–Henseleit solution of, in mM, NaCl 118.5; KH_2_PO_4_ 2.5; KCl 4.7; MgSO_4_ 1.2; CaCl_2_ 2.5; NaHCO_3_ 25; glucose 11. The pH of the solution was adjusted to 7.40 with NaOH, conditions that allowed cannulation of the aorta with a metal cannula of suitable diameter (18 G). The cannulated heart was placed on the Langendorff system (EMKA Technology) and perfused retrogradely (perfusion is in the opposite direction of *in vivo* blood flow) through the coronary artery network with Krebs–Henseleit solution. The perfusion fluid was thermostated at 37°C using a water bath (GD120. Grant) and saturated with a carbogen-type gas mixture (95% O_2_ and 5% CO_2_).

The ECG recording electrodes (Ag-AgCl) were placed on the epicardium of the right atrium near the sinoatrial node and in opposition to the apex (lead II of the human ECG). The electrode array was connected to a standard ECG amplifier and an analog-to-digital converter (EMKA Technologies, France). The signal was digitized with a sampling rate of 1 kHz. The heart was stabilized 15 minutes before the start of recording. The parameters of the cardiac activity were recorded continuously by a computer system dedicated to the acquisition with the IOX 2.9.5.43 software (EMKA Technologies, France).

After 15 minutes of stabilization in standard perfusion condition (Krebs–Henseleit), *H. bonariensis* extract was continuously perfused in increasing doses (1.56; 3.125; 6.25; 12.5; 25; 50 mg/ml) every 3 minutes. After each range, the heart was flushed with Krebs–Henseleit to measure reversibility.

### 2.4. Test of *H. bonariensis* Extract on Isolated Guinea Pig Auricles

The test of the effects of the extract on the isolated auricles of guinea pigs was carried out according to the method of Bonnah et al. Four male guinea pig was anesthetized as before. The heart was isolated as quickly as possible and placed in a physiological Krebs–Henseleit solution. The two atria were separated from the ventricles. The ends of the atria taken together were attached with wires. The preparation was then mounted in an isolated organ tank maintained at 37°C containing Mac Ewen's solution. Organ contraction was recorded using LabChart V6 software (ADI Instruments). After stabilization for 30 min, volumes of extract corresponding to final concentrations of 0.05, 0.1, 0.2, 0.4, and 0.8 mg/ml of *H. bonariensis* extract were injected into the vessel every 10 min, and the effects were recorded.

### 2.5. Testing of *H. bonariensis* Extract on Human Cardiac Sodium Current I Nav1.5

Recording of sodium current (INav1.5) was performed on human cardiac Nav1.5 channels transiently expressed in HEK 293 cells. Cells were cultured in Dulbecco's modified Eagle's medium (DMEM, HyClone, Logan, UT, USA) and 10% (v/v) fetal bovine serum (FBS, Gibco, Gaithersburg, MD, USA) in a humidified atmosphere at 37°C with 5% CO_2_-enriched air. Cells were subcultured every 2-3 days using brief trypsin treatments and seeded on 35 mm kneaders for 24–48 hours before patch-clamp experiments.

For I Nav1.5 recording, we used a pipette solution containing the following (in mmol/L): 10.0 NaCl, 10.0 CsCl, 120.0 CsF, 1.0 MgCl_2_, 1.0 CaCl_2_, 10.0 EGTA, and 10.0 HEPES (pH adjusted to 7.2 with CsOH). We also used an extracellular bath solution with the composition (in mmol/L) of 30.0 NaCl, 110.0 choline chloride, 5.0 CsCl, 1.2 MgCl_2_, 2.0 CaCl_2_, 10.0 HEPES, and 10.0 glucose (pH adjusted to 7.4 with NaOH).

Cells on coverslips were transferred to a continuously perfused recording chamber mounted on the stage of an inverted microscope and perfused with 10 ml of bath solution at a flow rate of approximately 1 ml/min for electrophysiological recording. The current signal was filtered at 5 kHz and sampled at 10 kHz. The capacitance and resistance of the entire cell were compensated, and leakage currents were subtracted. The currents were recorded 5 min after the whole-cellpatch-clamp configuration was completed.

Sodium currents were generated by clamping the cell membrane from a holding potential of −120 mV to potentials ranging from −100 mV to 40 mV for 50 ms in 10 mV increments, with 3-second intervals between stimuli. Cells were superfused with vehicle until stabilization and then with 0.5 mg/ml *H. bonariensis* extract (*n* = 6) until steady state, followed by a washout period with vehicle.

Fast and slow decay time constants were obtained by fitting the current traces with a double exponential equation. The voltage dependence of activation was determined from the relative membrane conductance versus potential using the formula GNa = INa/(Vm − Vrev), where GNa is the peak conductance and INa is the peak sodium current for the test potential Vm. Vrev is the estimated sodium current reversal potential obtained by extrapolating the current-voltage relationship. The resulting sodium ion conductance was normalized to the maximum response for each cell. The activation data were fitted with Boltzmann's equation: G/Gmax = 1/(1 + exp((V − V_1/2_)/*k*)), where Gmax represents the maximum conductance and V_1/2_ and *k* represent the half-maximum activation voltage and Boltzmann slope coefficient, respectively.

### 2.6. Testing of the Extract on the Human Cardiac Pacemaker Current If (“Funny Current”)

Recording of the If current was performed on the Chinese hamster ovary (CHO) cell-derived stable line expressing the HCN1 gene encoding the *f* channel. CHO cells were grown in DMEM (Dulbecco's Modified Eagle's Medium) culture medium supplemented with 1% antibiotics (100 IU/mL penicillin-G-Na; 50 IU/mL streptomycin sulfate), 1 nM insulin, and 10% fetal calf serum. Cells were seeded on 35 mm Petri dishes precoated with laminin (Invitrogen) and maintained at 37°C in a 5% CO_2_ atmosphere. Seeding density was approximately 2.0 × 104 cells/cm^2^, allowing cells to reach confluence in 2 to 3 days. The cell cultures were reseeded after trypsinization. Patch-clamp experiments were performed within 24 hours of culture progression.

We used Tyrode's extracellular bath solution (in mM) of NaCl 140; KCl 25; CaCl_2_ 1; MgCl_2_ 1.2; D-glucose 5.5; HEPES-NaOH 5 (pH = 7.4); BaCl_2_ 1; MnCl_2_ 2; and 4 aminopyridine 0.5. The intrapipette solution is composed of (in mM) KCl 40; NaCl 10; MgATP 2; GTP 0.3; CaCl_2_ 1; EGTA 11; HEPES-KOH 10 (pH = 7.2). 10 *µ*M cAMP was added to the pipette solution to amplify the current. Intracellular diffusion of cAMP is monitored immediately after membrane rupture under the patch clamp. After rupture of the seal and dialysis of the intracellular medium with cAMP, the If current increases rapidly to reach the steady state.

For current recording, the cells were superfused with Tyrode's solution. The cell membrane capacitance was measured by applying a low amplitude (±10 mV) voltage pulse from −40 mV. We applied a hyperpolarizing voltage pulse between −40 and −140 mV for 2.5 seconds from a holding potential of −40 mV to verify the presence of If current. Cells were superfused with vehicle until stabilization and then with *H. bonariensis* extract 0.5 mg/ml (*n* = 6) until steady state, followed by a washout period with vehicle.

## 3. Results

### 3.1. Effects of *H. bonariensis* Extract on the Electrocardiogram (ECG) of the Wistar Rat *In Vivo*

**Figure 1:**
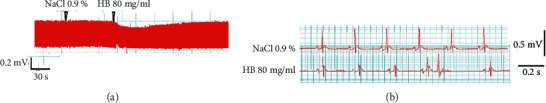
ECG recording in the presence of HB extract at 80 mg/kg. (a) The condensed recording. (b) The detail of the ECG waves.

Injection of increasing amounts of the hydroethanolic extract of the leaves of *H. bonariensis* caused effects on the recorded ECG, P wave, T wave, RR interval, and the heart rate of Wistar rats (Figure 1).  Table 1 shows that, at 80 mg/kg, the extract caused a significant decrease in P wave and T wave amplitudes of 29.06% and 41.54%, respectively, compared with the control. The extract did not impact either the QRS wave amplitude or the QT interval duration at the tested doses. However, from 20 mg/kg onwards, it induced a progressively significant increase in the duration of the RR interval as well as a significant proportional decrease in the heart rate of Wistar rats. The *H. bonariensis* extract thus induced a dose-dependent slowing down of the cardiac activity in Wistar rats.

### 3.2. Effects of *H. bonariensis* Extract on the Isolated Perfused Wistar Rat Heart (*In Vitro*)

**Figure 2:**
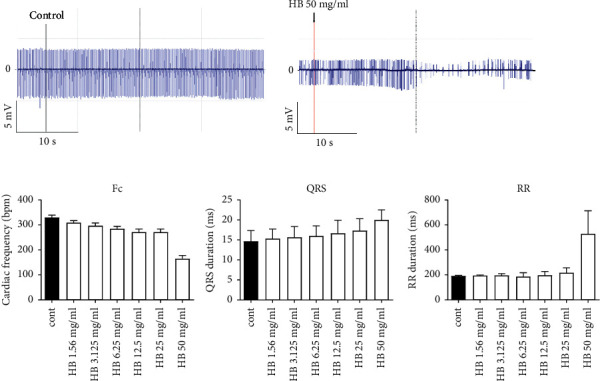
Effects of hydroethanolic extract of *H. bonariensis* leaves on the isolated perfused Wistar rat heart. Fc, QRS wave amplitude, and RR interval duration were analyzed. Perfusion of the extract caused a nonsignificant decrease in heart rate and a significant increase in RR interval at 50 mg/kg. n = 4. ^*∗*^*p* < 0.5 (2-way ANOVA+ Kruskal–Wallis test). Fc: heart rate; QRS: QRS wave amplitude; RR: RR interval duration; HB: *Hydrocotyle bonariensis*.

The effects of *H. bonariensis* extract were evaluated on some electrocardiogram parameters such as heart rate, QRS wave amplitude, and RR interval duration.  Figure 2 shows that the hydroethanolic extract of *H. bonariensis* leaves at 50 mg/kg causes a nonsignificant decrease in heart rate in the isolated perfused heart. In addition to a very slight nonsignificant increase in QRS amplitude, the extract, almost without effect from 1.56 to 25 mg/ml, causes a significant increase in RR interval duration from 192 ms (control) to 530.33 ms (at 50 mg/ml).

### 3.3. Effects of *H. bonariensis* Extract on Isolated Guinea Pig Atria

**Figure 3:**
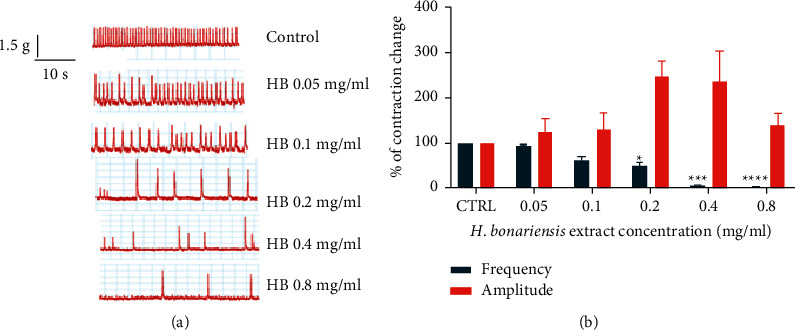
Effects of *H. bonariensis* extract on isolated Wistar rat atria. Injection of the extract into the organ tank caused a significant progressive decrease in atrial contraction frequency from 0.2 mg/ml. In contrast, contraction amplitudes were increased from the same concentration and then decreased from 0.4 mg/ml. *n* = 4. ^*∗*^*p* < 0.5; ^*∗*^*p* < 0.01; ^*∗∗∗*^*p* < 0.001; and ^*∗∗∗∗*^*p* < 0.0001 (two-way ANOVA+ Dunnett's test).

The hydroethanolic extract of *H. bonariensis* leaves causes, approximately one minute after its injection into the organ tank containing the isolated atria in full contraction, a progressively significant decrease in the frequency of contraction of the ears as the concentration of the injected extract increases. This decrease in the frequency of contraction is 97.64% at the concentration of 0.8 mg/kg compared to the control. This extract causes a significant increase in atrial contraction amplitude at the same concentrations from 0.2 mg/mL onward. The increase in amplitude which is 68.91% at 0.2 mg/ml compared to control reverses from 0.4 mg/ml (Figure 3).

### 3.4. Effects of *H. bonariensis* Extract on Human Cardiac Sodium Current I Nav1.5

**Figure 4:**
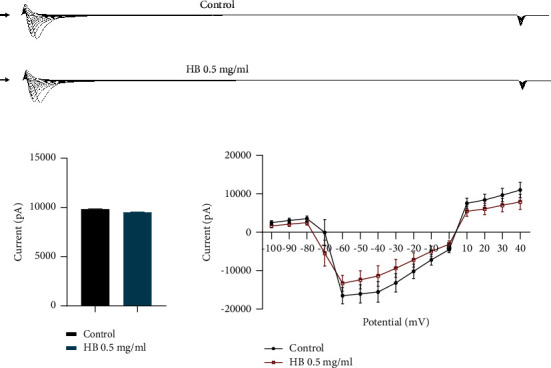
Effects of *H. bonariensis* extract on human cardiac sodium current I Nav1.5. Comparison of the amplitudes of cardiac sodium currents recorded on HEK 293-I Nav1.5 cells in control and 0.5 mg/kg *H. bonariensis*extract-treated conditions ((a) *t*-test + Mann–Whitney test). The effect of *H. bonariensis* extract on the current-voltage relationship of the I Nav1.5 channel was estimated by normalizing the current values plotted against the test potential in the absence and presence of the extract ((b) ANOVA+ Sidak's multiple comparisons test). No change in I Nav1.5 current is observed in the presence of the extract. *n* = 10.

We studied the effect of *Hydrocotyle bonariensis* leaf hydroethanolic extract (0.5 mg/mL) on INav1.5 sodium currents with transiently transfected HEK 293 cell lines (Figure 4). Cells were perfused with control until stabilization and then with *H. bonariensis* extract at 0.5 mg/ml until steady state, followed by a washout period with control. *H. bonariensis* extract at 0.5 mg/ml, as shown in Figure 4, has no effect on the amplitude of INav1.5 sodium current. Recording of the IV current in the presence of the 0.5 mg/ml *H. bonariensis* extract showed no difference from its recording under control conditions. Thus, the hydroethanolic extract of *H. bonariensis* has no effect on human cardiac sodium current.

### 3.5. Effects of *H. bonariensis* Extract on Human Cardiac Pacemaker Current If (“Funny Current”)

**Figure 5:**
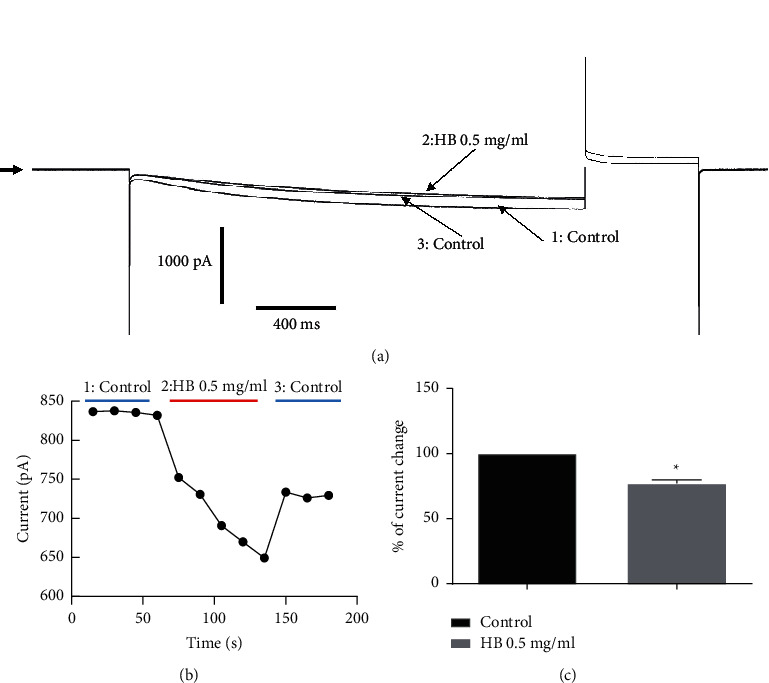
Effects of *H. bonariensis* extract on human cardiac If current. *n* = 6. Amplitude of If current in control and *H. bonariensis* extract 0.5 mg/kg treated condition. The extract induces a significant decrease in current amplitude. Variation is shown by the *t*-test + Mann–Whitney test (^*∗*^*p* < 0.05).

We evaluated the effect of hydroethanolic extract of *Hydrocotyle bonariensis* leaves (0.5 mg/ml) on If current with CHO cells expressing the human cardiac If channel.  Figure 5 shows that *H. bonariensis* extract at 0.5 mg/ml inhibits the If current to reach a steady state. This inhibitory effect is partially reversible and is estimated to be 24.55% (*p* < 0.05; *n* = 6).

**Figure 6:**
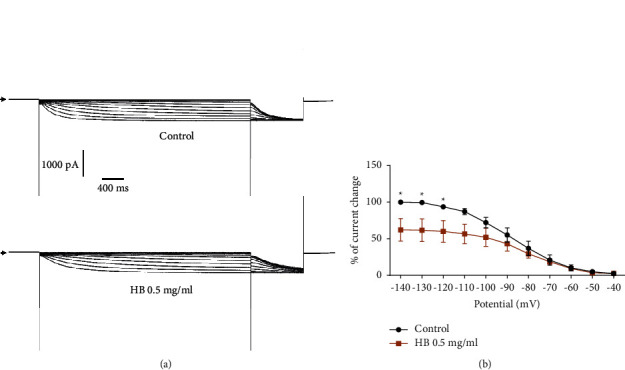
Activation curves from currents recorded on CHO-If cells by imposing the I/V stimulation protocol. *n* = 6. Normalized activation curve obtained from tail current traces as a function of prepotentials in control condition compared to *H. bonariensis* extract 0.5 mg/ml treatment (ANOVA+ Sidak's multiple comparisons test). ^*∗*^*p* < 0.05.

The effect of *H. bonariensis* extract on the current-voltage relationship of the If channel was estimated by normalizing the current values plotted against the test potential in the absence of the extract. Normalized currents were obtained from the maximum current amplitudes in response to depolarizing pulses in the range of −40 to −140 mV (Figure 6). Analysis of the activation curves indicates a significant decrease in maximum conductance of 37.82% at −40 mV.

## 4. Discussion

The first step in studying the effects of the *H. bonariensis* leaves extract on cardiac activity was to test this extract on the electrocardiogram of the Wistar rat. The results of this study showed that this extract transiently causes a significant decrease in heart rate, P wave, and T wave amplitude, followed by a significant increase in the duration of the RR interval. The T wave corresponds to the final phase of ventricular repolarization; thus, the decrease in its amplitude would imply that this extract would inhibit ventricular repolarization of the heart. The P wave materializes the atrial depolarization, and its decrease suggests that this extract would act on the muscarinic receptors. Indeed, the activation of muscarinic receptors is translated on the ECG by a decrease or an inversion of the P wave, a lengthening of the PR space, and an atrioventricular dissociation [11]. Similar effects with the aqueous extract of *Averrhoa carambola* on guinea pig hearts were observed [12], suggesting that the hydroethanolic extract contains cholinomimetic substances. The lengthening of the RR interval would translate slowing down of the cardiac contractions induced by this extract as it was shown by Adnet et al. [13]. This result is confirmed by the decrease in heart rate observed after intravenous administration of the hydroethanolic extract to normotensive rabbits. These results generally reflect a decrease in electrical activity and cardiac conduction, effects that could be induced by cholinomimetic substances. These effects could also be induced by the anticalcics contained in the extract. Indeed, anticalcics block L-typevoltage-dependent calcium channels in the vessels and heart, thus preventing the release of intracellular calcium necessary for contraction [9, 14–16].

The second step of the study of the effects of the extract on cardiac activity consisted of testing it on isolated organs such as the isolated perfused rat heart and the isolated guinea pig atria. Our results showed that the extract has a dose-dependent negative chronotropic effect as well as a transient positive inotropic effect on the mechanical activity of the atria. Inhibition of the atrial contraction rate by the extract could result in a decrease in electrical activity and cardiac conduction as explained above. This negative chronotropic action on the atria could also imply an interference of the bioactive compounds of the extract in the regulation of cardiac automatism. These effects are similar to those obtained by other authors who tested the effects of plants on isolated guinea pig atria [17, 18].

The third step in the study of the effects of the hydroethanolic extract of *H. bonariensis* leaves on cardiac function is cellular and was focused on human cardiac ionic electrophysiology. Indeed, the cardiac action potential is an electrical phenomenon finely regulated by modifications of the membrane voltage generated by the opening of various ion channels (sodium, calcium, chlorine, and potassium) present at the surface of cardiomyocytes. The transfer of ions across the membrane via these channels generates currents that can be measured by the patch-clamp technique. Therefore, cardiac electrical activity is the result of a balance of different ion flows that can also be modulated by the sympathetic and parasympathetic systems to allow adaptation of the heart rhythm [19]. The extract is tested on some of these ionic currents strongly involved in cardiac physiology and pathophysiology, such as sodium, potassium, and cardiac pacemaker currents.

We examined the effect of the extract on the cardiac subunit of the human sodium current (NaV1.5) in order to verify if this extract would influence the electrical activity of the heart via these channels. Divided into rapid (INa peak) and sustained (INaL) phases which respectively intervene in the excitability and duration of an AP (plateau phase), the sodium current (INa) is responsible for the initiation of action potentials (AP) and modulates its duration. It, therefore, plays a major role in cardiac excitability and electrical impulse conduction. Because of this, a change in the biophysical properties of INa during pathophysiological conditions can generate rhythm modulation [20, 21]. The results of our voltage-gated work on INa revealed that this extract has no influence on the cardiac subunit of sodium current. No effect is observed on either the amplitude or the current-voltage relationship of human cardiac sodium channels. The mechanism of action of this extract would not, therefore, involve the sodium channels.

In addition to the test on cardiac sodium current, we studied the impact of the hydroethanolic extract of *Hydrocotyle bonariensis* leaves on cardiac rhythmicity by evaluating its effects on the human cardiac pacemaker current If.

The If current, called “funny current,” modulating the heart rate, was also a target of our study of the effects of the extract of *H. bonariensis* on cardiac rhythmicity. This current activated by membrane hyperpolarization is particularly present in sinus cells with pacemaker activity (automatic and regulating the cardiac rhythm). It controls slow diastolic depolarization at the sinus node, exclusively regulating the heart rate [22]. In this study, we have shown that the extract of *H. bonariensis* at the concentration of 0.5 mg/ml significantly decreases the amplitude of the If current while decreasing the current-voltage relationship (curve IV). The extract reduces the conductance of the channels without altering their kinetics. The bioactive compounds in the extract would, therefore, partially block the If channel pore. The effect of the extract is partially reversible. This partial reversibility would be the binding of the bioactive compounds on different sites of one or more subunits of the If channel. Furthermore, it is plausible that these sites have a variable affinity towards the different components present in the extract, further explaining the partial reversibility. These results are similar to other studies performed on the pharmacology of If channels, leading to cardiac therapeutic molecules against stable angina and myocardial ischemia [23, 24]. This If current produced by specific If channels and playing an essential role in the depolarization of the sinus node is today the target of some therapeutic molecules. It is specifically and selectively inhibited by a benzocyclobutene compound called ivabradine. This is a bradycardia-inducing molecule that does not affect other currents present in the cells of the sinus node. The hydroethanolic extract of the leaves of *H. bonariensis,* like ivabradine, has the property of slowing down the heart rate by inhibiting the If current, the regulatory current of the cardiac pacemaker.

## 5. Conclusion

The hydroethanolic extract of the leaves of *Hydrocotyle bonariensis* has an overall inhibitory effect on cardiac activity. Cardioinhibition is one of the causes of the reduction of blood pressure figures due to the reduction of systolic ejection volume and heartbeat cycles. This would justify its use in the traditional management of hypertension.

In addition to the study of the effects of the extract of this plant on cardiac activity, the mammalian vascular system would be a complementary target to study its antihypertensive effects.

## Figures and Tables

**Table 1:** Effects of hydroethanolic extract of *H. bonariensis* leaves on the ECG of Wistar rats.

	P wave (%)	QRS wave (%)	T wave (%)	RR interval (%)	QT interval (%)	Heart rate (%)
Control	100	100	100	100	100	100
HB 10 mg/kg	99,65	92,14	103,72	118,47	110,58	80,48
HB 20 mg/kg	106,88	95,44	91,18	133,85^*∗∗*^	105,74	72,47^*∗∗*^
HB 40 mg/kg	98,98	88,77	93,87	131,54^*∗∗*^	111,50	66,77^*∗∗*^
HB 80 mg/kg	70,93^*∗*^	95,22	58,46^*∗∗∗*^	152,23^*∗∗∗∗*^	104,32	49,02^*∗∗∗∗*^

The amplitudes of the P, QRS, and T waves, the duration of the RR interval, and then the heart rate were analyzed. Infusion of the extract caused a significant decrease in P wave and T wave at 80 mg/kg and then heart rate and a significant increase in RR interval at 20, 40, and 80 mg/kg. *n* = 4. ^*∗*^*p* < 0.5; ^*∗∗*^*p* < 0.01; ^*∗∗∗*^*p* < 0.001; ^*∗∗∗∗*^*p* < 0.0001 (2-way ANOVA+ Kruskal–Wallis test). HR: heart rate; HB: *Hydrocotyle bonariensis.*

## Data Availability

The data used to support the findings of this study are included in the article.

## Additional Points

(i) *Hydrocotyle bonariensis* decreases heart rate in all tested EGC study models. (ii) *Hydrocotyle bonariensis* partially blocks human if current but has no effect on human cardiac INav1.5 current. (iii) Hy*drocotyle bonariensis* has a cardiomoderating effect.
